# Household costs among patients hospitalized with malaria: evidence from a national survey in Malawi, 2012

**DOI:** 10.1186/s12936-017-2038-y

**Published:** 2017-10-02

**Authors:** Ian Hennessee, Jobiba Chinkhumba, Melissa Briggs-Hagen, Andy Bauleni, Monica P. Shah, Alfred Chalira, Dubulao Moyo, Wilfred Dodoli, Misheck Luhanga, John Sande, Doreen Ali, Julie Gutman, Kim A. Lindblade, Joseph Njau, Don P. Mathanga

**Affiliations:** 1 Malaria Branch, Division of Parasitic Diseases and Malaria, Center for Global Health, Centers for Disease Control and Prevention, Atlanta, GA USA; 2 Malaria Alert Center, Malawi College of Medicine, Blantyre, Malawi; 3 National Malaria Control Programme, Malawi Ministry of Health, Lilongwe, Malawi; 4 World Health Organization, Lilongwe, Malawi

**Keywords:** Malaria, Household costs, Malawi, Inpatient malaria, Economic burden

## Abstract

**Background:**

With 71% of Malawians living on < $1.90 a day, high household costs associated with severe malaria are likely a major economic burden for low income families and may constitute an important barrier to care seeking. Nevertheless, few efforts have been made to examine these costs. This paper describes household costs associated with seeking and receiving inpatient care for malaria in health facilities in Malawi.

**Methods:**

A cross-sectional survey was conducted in a representative nationwide sample of 36 health facilities providing inpatient treatment for malaria from June–August, 2012. Patients admitted at least 12 h before study team visits who had been prescribed an antimalarial after admission were eligible to provide cost information for their malaria episode, including care seeking at previous health facilities. An ingredients-based approach was used to estimate direct costs. Indirect costs were estimated using a human capital approach. Key drivers of total household costs for illness episodes resulting in malaria admission were assessed by fitting a generalized linear model, accounting for clustering at the health facility level.

**Results:**

Out of 100 patients who met the eligibility criteria, 80 (80%) provided cost information for their entire illness episode to date and were included: 39% of patients were under 5 years old and 75% had sought care for the malaria episode at other facilities prior to coming to the current facility. Total household costs averaged $17.48 per patient; direct and indirect household costs averaged $7.59 and $9.90, respectively. Facility management type, household distance from the health facility, patient age, high household wealth, and duration of hospital stay were all significant drivers of overall costs.

**Conclusions:**

Although malaria treatment is supposed to be free in public health facilities, households in Malawi still incur high direct and indirect costs for malaria illness episodes that result in hospital admission. Finding ways to minimize the economic burden of inpatient malaria care is crucial to protect households from potentially catastrophic health expenditures.

## Background

Despite significant reductions in malaria burden in recent years, malaria remains a major cause of morbidity and mortality in Malawi. With an estimated 3.3 million malaria cases and 7200 deaths in 2015, malaria accounts for 34% of all outpatient consultations and remains the leading cause of death among children under 5 years old [1, 2]. Prompt case management of malaria with an effective first-line medication is a cornerstone of malaria control policy in Malawi. Correct management of uncomplicated *Plasmodium falciparum* malaria with artemisinin-based combination therapy (ACT), and management of severe malaria with parenteral artesunate or quinine contributes to reducing malaria mortality [1, 3]. Likewise, early diagnosis and treatment of severe malaria is associated with significantly reduced mortality rates [4]. Although the importance of early detection and treatment of malaria episodes is widely recognized, prompt diagnosis and effective treatment remains limited in Malawi. In the 2014 malaria indicator survey (MIS), a nationally representative household survey, only 59% of children with recent history of fever sought care and 42% received an antimalarial [5].

The perceived and actual financial costs of care for malaria are important barriers to accessing prompt and effective malaria treatment and these costs can be economically catastrophic for many households [6–8]. In a review of 65 studies conducted in over 15 countries in sub-Saharan Africa including Malawi, mean direct household costs for the treatment of uncomplicated malaria ranged from $0.30 ($0.34 in constant 2012 dollars) in Tanzania to $6.54 ($6.97 in constant 2012 dollars) in Mozambique [8, 9] (pers. comm., Njau and McFarland). While information on the household costs of severe or inpatient malaria is less available, studies have observed costs between $6.40 ($8.82 in constant US dollars) in Ghana to $17.20 ($22.30 in constant US dollars) in Sudan [10, 11]. Given that more than half of the population of Malawi lives below the national poverty level, and 71% lives on less than $1.90 a day [12], the cost of malaria treatment may be prohibitive for many households, potentially resulting in delayed care seeking and inadequate treatment [13]. Despite negotiated prices and subsidies, ACT remains more expensive than previous first-line medications such as sulfadoxine-pyrimethamine or chloroquine [14]. When direct payments for malaria care are removed, patients are significantly more likely to seek care for malaria episodes [15].

Few studies have examined the household costs of uncomplicated malaria and febrile illness treatment in Malawi and there are virtually no studies that have evaluated the household costs of inpatient malaria treatment. One study in 2004 found that mean reported direct costs per febrile episode amount to $1.05 (or $1.28 in constant US 2012 dollars) in urban areas compared to $0.21 ($0.26 in constant 2012 dollars) in rural areas [16]. These costs are particularly burdensome for very low income households; an earlier study found the total costs of malaria treatment consume almost 30% of annual income in very low income households, compared to 2% of income in low to high income households [17]. Many Malawians experience multiple malaria episodes per year [18], which multiplies the economic burden of malaria care on households. Direct costs associated with treatment-seeking vary with distance to health facilities; another study found harder-to-reach households incurred higher costs per fever episode ($5.24; $5.52 in constant 2012 dollars) compared to those who lived nearer to health facilities ($4.46; $4.70 in constant 2012 dollars) [19]. However, there are little to no data about the household costs of malaria illnesses requiring inpatient management in Malawi.

In order to ensure equitable access to malaria treatment, especially among the most vulnerable populations, it is essential to understand the household costs of inpatient management of malaria. This study examines the direct and indirect household costs associated with malaria illness episodes resulting in inpatient treatment in Malawi, as well as the most important drivers of those costs. This analysis was conducted within a larger study of inpatient malaria case management at public and private nonprofit mission health facilities in Malawi. The study assessed different aspects of facility readiness to manage malaria cases, including availability of antimalarial and other supplies, availability of malaria diagnostic services, health worker training and adherence to national guidelines for the diagnosis and treatment of severe malaria. Details of this study are published elsewhere [20].

## Methods

### Study setting

Malaria is endemic throughout Malawi, where the entire population of 17.2 million is considered at risk [1]. All three regions of the country experience stable, high levels of transmission, although there is significant seasonal variation, with higher transmission during the rainy season from November to April in most parts of the country [21]. The most recent MIS (2014) found that 33.2% of children aged 6–59 months were parasitaemic by microscopy [5]. Artemether–lumefantrine (AL) is the recommended first-line treatment for uncomplicated malaria while intravenous quinine or artesunate are recommended for severe malaria episodes. Oral quinine is recommended for pregnant women in their first trimester and children under 5 kg [22].

Health care is delivered free of charge at Ministry of Health (MoH) facilities, whereas Christian Health Association of Malawi (CHAM) facilities receive some government subsidies but charge a nominal fee [19]. An essential health package (EHP), including basic care for malaria and other common infectious diseases, is delivered free of charge at both types of facilities. Primary care is offered by a cadre of village clinics staffed by community health workers (CHWs), health centres, and hospitals, whereas secondary care is typically provided at rural and district hospitals [22].

### Sampling and eligibility

A nation-wide, cross-sectional survey was conducted in 36 health facilities from June through August of 2012. The sampling strategy is described in more detail elsewhere [20]. Briefly, these facilities were systematically selected with equal probability from a list of all 91 MoH and CHAM facilities in Malawi that admit patients for severe malaria. Private, for profit health clinics, which typically do not have inpatient care capacity, were excluded from the sampling frame. The eligible facilities were listed first in order of region, then in order of management (MoH or CHAM), and lastly in order of hospital type (district, community, or other) prior to systematic random selection.

Patients were eligible to participate in the inpatient interview if they had been admitted with any malaria diagnosis, or admitted with another diagnosis but started on an anti-malarial treatment, at least 12 h prior to the initial arrival of the study team. Patients who were to be discharged on the day of the interview or had sought care at another facility before hospital admission were eligible to provide information on household costs associated with their present malaria episode. Caregivers were asked to respond for patients aged < 12 years or those unable to speak for themselves.

### Data collection and outcome definitions

Trained interviewers used Dell Axim X51 personal digital assistants (PDAs) to collect demographic and cost information from eligible patients or their caregivers. The questionnaires were programmed in visual CE (Syware, Boston MA). Questionnaires elicited demographic information for patients and their caregivers (e.g., age and sex) and information about the patient’s current malaria episode and treatment seeking (e.g., number of days ill, distance from a patient’s home to the present facility). Information on household assets and economic characteristics was also elicited. Patients were then asked to provide cost information for their visit to the current facility as well as any previous visits to health facilities for the current malaria episode. Total costs associated with malaria episodes resulting in inpatient admission was considered the primary outcome, while direct and indirect costs were secondary outcomes.

### Costing approach

Direct medical and non-medical costs were estimated using an ingredients-based approach in which the patient or caregiver was asked to recollect the costs incurred for individual items throughout their illness episode [23, 24]. Direct costs were subdivided into two categories: (i) medical costs, and (ii) non-medical costs. Direct medical costs included costs for registration, consultation, supplies, hospital bed, and medications. Direct non-medical costs included travel, meals, and accommodation.

Indirect costs were estimated using a human capital approach [25, 26]. For patients 14 years and older, productive time lost was estimated as the total number of days spent travelling to and from the facility, waiting for admission, and days spent in care as well as time spent ill prior to seeking care for their current malaria episode. Productive lost time for accompanying caregivers was similarly estimated as the total number of days spent accompanying the patient while travelling to and from the facility, waiting for the patient to be admitted, and accompanying the patient while he or she received care as well as time spent caring for the patient while they were ill prior to seeking care for their current malaria episode. Given the high level of self or informal employment in the country, the national minimum daily wage (317 Malawi Kwacha (MWK), or about USD $1.30, in 2012 [27]) was used to value the cost of time lost. Associated costs due to lost productivity were estimated as the product of total time lost and the minimum wage. For children below the minimum age for employment in Malawi (< 14 years old), only productivity losses for their accompanying caregivers were considered [28]. Additionally, lost school fees due to absenteeism were not included. Total household costs associated with each illness episode resulting in inpatient management were obtained by summing total direct and indirect costs for each health facility visit over the course of the entire illness episode.

### Statistical analysis

All statistical analyses were performed using SAS version 9.4 (SAS institute, Cary, NC) and STATA IC/14 (Stata-corp LP, Texas, USA). Frequencies and cross tabulations on patient demographics and mean and median direct and indirect costs were calculated using survey procedures with a cluster statement to account for clustering of individual observations at the health facility level. Household wealth index was calculated from household characteristic and asset data using principal component analysis [29]. Households were then divided into household wealth tertiles (poorest, middle and least poor) according to their wealth index score. Given the highly-skewed nature of the cost data (see below for more details on model choice and justification), generalized linear models (GLM) using maximum likelihood estimation were fit to examine bivariate associations between individual independent variables and total household costs. Independent variables included patient sex, age, household wealth tertile, rural residence, type of health facility and facility management (CHAM or MoH), whether the patient sought care prior to coming to the current facility, the length of the hospital stay, and the distance from the patient’s home to the health facility.

Standard diagnostics showed that the primary outcome (household costs) was highly skewed to the right and heteroskedastic (Fig. 1a, b), precluding the use of parametric tests. Generalized linear models (GLMs) were therefore used to analyse cost data while taking into account the non-normal distribution of the data [30]. The distribution (F) of the dependent variable and the link function (g) describing how independent variables are functionally related to the dependent variable need to be specified in GLMs [31]. To select appropriate distribution and link functions for the primary study outcome, the modified Parks test was used [32], and a log link with Gamma distribution provided the best fit for the data.

**Fig.s 1:**
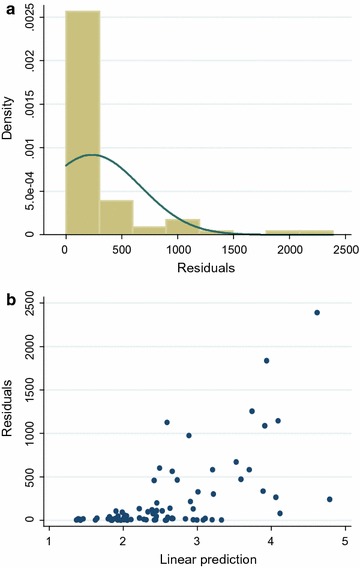
Regression diagnostics for ordinary least squares on household costs ($). **a** Distribution of squared residuals. **b** Scatter of residuals vs. fitted values, showing increasing variations of points as predicted values increase, suggestive of heteroskedasticity

The empirical GLM took the form:

$$g\left( {\mu_{i} } \right) = \beta_{0} + \beta_{{1{\text{i}}}} X_{i} , y_{i} \sim F$$

where $\mu_{i}$ denotes the dependent variable of interest (household costs) for every unit (case treated for inpatient malaria), and X_i_ is a vector of independent factors known or presumed to influence the household costs as outlined above. Two models were run; in the full model, all independent factors were included. In the restricted model, a backward stepwise regression was used, beginning with the full model, and retaining only independent factors with corresponding p values < 0.05. Robust standard errors clustered at the health facility level were generated. The exponential of coefficients (β_i_) in GLMs with a log link are interpreted as the ratio of arithmetic means between groups [30].

### Ethical considerations

Ethical approval from both the US centers for disease control and prevention institutional review board (Atlanta, GA USA) and the university of Malawi college of medicine research and ethics committee (Blantyre, Malawi) was granted prior to study implementation. Written informed consent was obtained from patients 18 years and older, or caregivers for incapacitated patients or patients less than 18 years prior to data collection. Patients aged 7–18 years and not incapacitated were asked to provide written assent as well.

## Results

### Characteristics of survey respondents

Out of 100 patients who were eligible to provide cost information, 80 (80%) provided cost information about their current malaria episode and were included in this analysis (Table 1). The 20 patients who did not provide cost information were significantly older [mean 30.7; median 27.5, interquartile range (IQR) 4.5–55.5] than those who did provide cost information (*p* = 0.01), but otherwise did not differ based on demographics, illness, and treatment seeking characteristics. Among patients who did provide cost information, the mean age was 13 years (median 4.9; IQR 2–17.5); 31 (39%) of the patients were under 5 years old, 46 (57%) were 5 years or older, and 3 (4%) were of unknown age. Approximately half (54%) were female, and the majority (94%) lived in rural areas.

**Table 1:** Characteristics of patients who provided information about household costs for management of malaria episodes resulting in hospitalization in Malawi, by age group, 2012 (N = 80)

	< 5 years	≥ 5 years	Total %
	n = 31 (%)	n = 46 (%)	n = 80
Age in years, median (IQR)	1.6 (1.0, 2.7)	12.0 (5.9, 30.6)	4.9 (2.0, 17.5)
Sex			
Male	14 (45.7)	22 (47.8)	37 (46.3)
Female	17 (54.8)	24 (31.2)	43 (53.8)
Residence			
Rural	30 (96.8)	42 (91.3)	5 (6.25)
Urban/small town	1 (3.22)	4 (8.7)	75 (93.8)
Wealth tertile			
Least poor	5 (16.1)	20 (43.5)	26 (32.5)
Middle	11 (35.5)	15 (32.6)	26 (32.5)
Poorest	15 (48.4)	11 (23.9)	28 (35.0)
Health facility management			
MoH	20 (64.5)	24 (52.2)	46 (57.5)
CHAM	11 (35.5)	22 (47.8)	34 (42.5)
Health facility type			
District/central	16 (51.6)	18 (39.1)	36 (45.0)
Other hospital type	10 (32.3)	22 (47.8)	33 (41.3)
Community/rural	5 (16.1)	6 (13.1)	11 (13.8)
Days in hospital, median (IQR)	1.4 (0.7, 2.1)	2.0 (0.6, 2.7)	1.5 (0.6, 2.6)
Days of illness, median (IQR)	1.8 (0.7, 3.5)	3.5 (1.3, 7.5)	2.8 (0.9, 5.8)
Sought care prior			
Yes	19 (61.3)	40 (87.0)	60 (75.0)
No	12 (38.7)	6 (13.0)	20 (25.0)
Distance, home to facility (km)			
< 5	13 (41.9)	23 (50.0)	36 (45.0)
≥ 5	18 (58.1)	23 (50.0)	44 (55.0)
Travel time to and from facility in days, median (IQR)	0.14 (0.1, 0.2)	0.16 (0.0, 0.2)	0.15 (0.1, 0.2)

*CHAM* Christian Health Association of Malawi, *MoH* Ministry of Health, *IQR* interquartile range

Patients reported being ill for a mean of 7.4 days (median 2.8; IQR 0.9–5.8) prior to seeking care at the current facility, and spent a mean of 2.5 days admitted to the health facility (median 1.5; IQR 0.0–12.0). A total of 60 (75%) patients had sought care at another facility prior to admission at the study facility, and 26 (32.5%) patients were discharged on the day of the study. Patients lived a mean distance of 10.2 kilometres (median 4.4; IQR 1.4–10.0) away from the health facility, and more than half (55%) lived 5 or more kilometres away. Patients travelled for an average of 0.2 days (median 0.1; IQR 0.0–1.7) to and from the facility in order to access care.

### Total household costs

The mean household cost of malaria episodes resulting in inpatient admission, including direct and indirect costs, was $17.48 (median $9.78; IQR $3.66–$23.56). In bivariate analysis (Table 2), household costs averaged $23.43 (median $17.00; IQR $7.97–$28.74) for patients who visited CHAM facilities compared to $13.09 (median $6.59; IQR $2.08–$14.99) for those who visited MoH facilities (*p* = 0.06). Children under 5 years incurred a mean total cost of $9.74 (median $7.15; IQR $2.09–$13.09) compared to $22.86 (median $14.23; IQR $4.94–$33.57) for those 5 years and older (*p* < 0.01). Patients from the least poor wealth tertile had higher mean costs at $28.06 (median $17.00; IQR $6.49–$42.62) than those in the middle tertile (mean $9.82; median $7.58; IQR $2.13–$13.34) and poorest tertile (mean $14.39; median $8.05; IQR $2.20–$23.56) (*p* < 0.01). Household wealth was not associated with the likelihood of patients attending a CHAM vs. MoH facility (*p* = 0.14).

**Table 2:** Bivariate predictors of total household cost for the management of malaria episodes resulting in hospitalization in Malawi, 2012 (N = 80)

	n	Mean	Median	IQR		*p* value
Total costs	80	$17.48	$9.78	$3.66	$23.56	
Facility ownership						
CHAM	34	$23.43	$17.00	$7.97	$28.74	0.06
MOH^r^	46	$13.09	$6.59	$2.08	$14.99	
Facility type						
District hospital	36	$13.32	$6.39	$2.01	$13.09	< 0.01
Rural/community/other	11	$13.50	$9.30	$4.51	$17.61	
Cost by age (years)						
<5	31	$9.74	$7.15	$2.09	$13.32	< 0.01
≥5^r^	46	$22.86	$14.23	$4.94	$33.57	
Sex						
Male	37	$13.08	$7.35	$2.03	$16.27	0.12
Female^r^	43	$21.27	$13.05	$6.16	$27.71	
Wealth tertile						
Least poor	26	$28.47	$17.00	$6.49	$42.62	< 0.01
Middle	26	$9.82	$7.58	$2.13	$13.34	
Poorest^r^	28	$14.39	$8.05	$2.20	$23.56	
Duration of hospital stay						
Long (≥ 2 days)	26	$24.53	$19.38	$12.67	$30.47	0.16
Short (< 2 days)^r^	16	$14.76	$6.39	$6.98	$14.23	
Sought care prior						
Yes	60	$19.00	$9.39	$3.29	$17.00	0.02
No^r^	20	$12.94	$9.78	$3.66	$26.46	
Distance from home to HF (km)						
< 5	36	$15.78	$8.05	$3.29	$17.00	0.54
≥ 5^r^	44	$18.88	$13.01	$3.66	$26.46	
Residence						
Rural	75	$15.27	$8.60	$3.02	$19.47	< 0.01
Urban/small town^r^	5	$50.68	$26.57	$22.04	$63.10	

*MoH* Ministry of Health, *CHAM* Christian Health Association of Malawi, *HF* health facility

^r^Reference

Patients from rural residences incurred lower costs at a mean of $15.03 (median $8.60; IQR $3.02–$19.47) compared to $50.68 (median $26.57; IQR $22.04–$63.10) for patients from urban or small town residences (*p* < 0.01). Patients who had sought prior care for their present malaria episode had significantly higher mean costs at $19.00 (median $9.39; IQR $3.29–$17.00) compared to $12.94 (median $9.78, IQR $3.66–26.46) for patients who had not sought prior care (*p* = 0.02). Mean household costs were $11.56 per night admitted (median $6.78; IQR $3.42–$12.09). Mean costs for patients who were discharged the day of the interview were $13.89 (median $13.89; IQR $6.68–$16.95), compared to $16.05 (median $5.85; IQR $1.01–$16.83) for patients who were not discharged the day of the interview (*p* = 0.62).

Multivariate GLM regression was used to determine patient and facility characteristics associated with total household costs per malaria episode (Table 3). In the restricted model, and controlling for all other variables, household costs for management of malaria illness resulting in admission in CHAM facilities were 52% higher than at MoH facilities (*p* < 0.01). Household costs for children under 5 years were 40% lower than for patients 5 years and older (*p* < 0.01). Household costs were 40% lower for patients that lived < 5 km away from the health facility compared to those who lived 5 km or greater away (*p* < 0.01). Each day in the hospital was associated with a 2% increase in overall household costs (*p* = 0.03). Costs for households in the wealthiest tertile were 82% higher than costs for households in the middle and poorest tertiles (*p* < 0.01).

**Table 3:** GLM regression results for the predictors of the total household cost of malaria episodes resulting in hospitalization in Malawi, 2012 (N = 80)

	Coefficient (β)	Robust standard error	Ratio of arithmetic means^a^	95% confidence interval		*p* value
*Full model*						
Sex (female vs. male)	0.48	0.47	1.62	0.65	4.06	0.302
Age < 5 years vs. ≥ 5 years	−0.51	0.08	0.60	0.51	0.71	< 0.01
Wealth tertile, middle vs. least poor and poorest	−0.54	−4.18	0.59	0.46	0.75	< 0.01
Wealth tertile, poorest vs. least poor and middle	−0.45	−2.58	0.64	0.45	0.90	0.01
CHAM vs. MoH	0.40	0.05	1.49	1.35	1.65	< 0.01
Rural vs. urban residence	−0.20	0.27	0.82	0.48	1.38	0.456
Days in hospital	0.02	0.00	1.02	1.01	1.03	< 0.01
Distance to health facility (< 5 km vs. ≥ 5 km)	−0.64	0.26	0.53	0.31	0.88	0.015
*Restricted model*						
Age < 5 years vs. ≥ 5 years	−0.51	0.09	0.60	0.51	0.71	< 0.01
Wealth tertile, least poor vs. middle and poorest	0.60	0.08	1.82	1.57	2.11	< 0.01
CHAM vs. MoH	0.42	0.06	1.52	1.36	1.70	< 0.01
Days in hospital	0.02	0.01	1.02	1.00	1.04	0.03
Distance to health facility (< 5 km vs. ≥ 5 km)	−0.50	0.05	0.61	0.55	0.67	< 0.01

*MoH* Ministry of Health, *CHAM* Christian Health Association of Malawi

^a^Exponentiated coefficient β_i_; multiplicative effect of variable on total household cost

### Direct household costs

The mean direct cost incurred by patients admitted for malaria episodes was $7.59 (median $7.59; IQR $0.91–$9.51) (Table 4). Costs differed significantly by facility management, averaging $5.59 (median $2.15; IQR $0.69–$6.40) at MoH health facilities compared to $10.28 at CHAM facilities (median $4.75; IQR $1.65–$13.67) (*p* < 0.05) (Table 5). Direct costs for patients under 5 years (mean $5.30; median $2.15; IQR $1.29–$7.50) were not significantly different from those for patients 5 years and over (mean $9.43; median $4.02; IQR $0.64–$13.87) (*p* = 0.06), and no other predictors were found to be significant. Direct medical costs averaged $2.39 (median $0.00; IQR $0.00–$2.62), with medications accounting for almost all direct medical costs (mean $2.38; median $0.00; IQR $0.00–$2.62). These direct medical costs differed significantly by facility ownership, averaging $1.06 (median $0.00; IQR $0.00–$0.82) at MoH health facilities compared to $4.19 (median $2.13; IQR $0.00–$5.85) at CHAM facilities (*p* = 0.01). Direct non-medical costs averaged $5.20 (median $1.89; IQR $0.11–$6.22); these averaged $4.54 (median $1.92; IQR $0.32–$4.94) for MoH facilities and $6.09 (median $1.83; IQR $0.00–$6.04) at CHAM facilities (*p* = 0.42). Meals and travel accounted for the largest proportion of direct non-medical costs and were the two most substantial contributors to overall direct costs, but were not significantly different by CHAM vs. MoH management type.

**Table 4:** Direct household costs in USD for management of malaria episodes resulting in hospitalization at health facilities in Malawi, 2012 (N = 80)

	Mean	Median	IQR	
Total direct costs	$7.59	$3.12	$0.91	$9.51
Direct medical costs	$2.39	$0.00	$0.00	$2.62
Medications	$2.38	$0.00	$0.00	$2.62
Direct non-medical costs	$5.20	$1.89	$0.11	$6.22
Travel to and from HF	$3.70	$1.04	$0.00	$3.23
Accommodation	$0.05	$0.00	$0.00	$0.00
Meals	$4.42	$1.94	$0.61	$5.81

*HF* health facility, *IQR* interquartile range

**Table 5:** Direct household costs in USD for management of malaria episodes resulting in hospitalization at health facilities in Malawi, by facility type and age, 2012 (N = 80)

	MoH^a^				CHAM^b^				*p* value
	Mean	Median	IQR		Mean	Median	IQR		
Total direct costs	$5.59	$2.15	$0.69	$6.40	$10.28	$4.75	$1.65	$13.67	0.05
Direct medical costs	$1.06	$0.00	$0.00	$0.82	$4.19	$2.13	$0.00	$5.85	0.01
Medications	$1.06	$0.00	$0.00	$0.82	$4.16	$1.95	$0.00	$5.85	0.01
Direct non-medical costs	$4.54	$1.92	$0.32	$4.94	$6.09	$1.83	$0.00	$6.04	0.42
Travel to and from HF	$3.29	$1.65	$0.00	$3.06	$4.26	$0.36	$0.00	$4.02	0.53
Accommodation	$0.09	$0.00	$0.00	$0.00	$0.00	$0.00	$0.00	$0.00	0.31
Meals	$3.80	$1.65	$0.61	$4.02	$5.20	$2.20	$0.37	$5.73	0.42
	**Patients < 5 years** ^**c**^				**Patients ≥ 5 years** ^**d**^				
Total direct costs	$5.30	$2.15	$1.29	$7.50	$9.43	$4.02	$0.64	$13.87	0.06
Direct medical costs	$2.00	$0.00	$0.00	$2.13	$2.79	$0.00	$0.00	$3.17	0.40
Medications	$2.00	$0.00	$0.00	$2.13	$2.79	$0.00	$0.00	$3.17	0.42
Direct non-medical costs	$3.30	$1.56	$0.21	$4.30	$6.63	$2.10	$0.00	$6.40	0.06
Travel to and from HF	$2.65	$0.82	$0.00	$2.74	$4.58	$1.65	$0.00	$3.84	0.22
Accommodation	$0.00	$0.00	$0.00	$0.00	$0.05	$0.00	$0.00	$0.54	0.39
Meals	$2.52	$0.62	$0.55	$2.56	$5.52	$2.12	$0.98	$6.86	0.13

*MoH* Ministry of Health, *CHAM* Christian Health Association of Malawi, *HF* health facility, *IQR* interquartile range

^a^N = 46, ^b^ N = 31, ^c^ N = 31, ^d^ N = 46

### Indirect costs

Overall, each malaria episode resulted in a mean loss of 8.5 productive days (median 4.1; IQR 0.0–11.1), including lost productive days for both patients and their caregivers. This was associated with a mean lost productivity cost of $9.90 per episode (median $4.74; IQR $0.00–$12.84) (Table 6). While patients under 14 did not themselves incur any indirect costs due to lost days of work, the productivity lost by their accompanying adult caregivers was calculated for their illness episode. When caregiver time is included, total lost productivity time for patients under 14 years was 4.1 productive days (median 0.0; IQR 0.0–6.2), which was considerably smaller than 19.6 days for patients 14 years and older (median 13.9; IQR 7.1–20.3). This was associated with an average lost productivity cost of $4.73 (median $0.00; IQR $0.00–$12.84) for patients under 14 years compared to $22.70 (median $16.08; IQR $8.23–$23.49) for patients 14 years and older.

**Table 6:** Indirect household costs in USD for the management of malaria episodes resulting in hospitalization in Malawi, 2012 (N = 80)

	Productive days lost due to malaria episode					Associated productivity costs			
	n	Mean	Median	IQR		Mean	Median	IQR	
*Total indirect costs (combined patient and caregiver costs)*	80	8.5	4.1	0.0	11.1	$9.90	$4.74	$0.00	$12.84
Total indirect costs < 14 years	57	4.1	0.0	0.0	6.2	$4.73	$0.00	$0.00	$7.13
Total indirect costs ≥ 14 years	23	19.6	13.9	7.1	20.3	$22.70	$16.08	$8.23	$23.49
Lost productivity for patients≥ 14 years	23	10.4	6.1	5.2	9.2	$15.89	$8.31	$5.97	$16.50
Lost productivity prior to the current admission^a^	23	12.0	6.5	2.9	12.2	$13.97	$7.54	$3.33	$14.11
Lost productivity during current admission^b^	23	1.7	0.7	0.1	2.4	$1.92	$0.78	$0.13	$2.79
Lost productivity for caregivers	80	9.2	7.1	4.3	11.1	$10.65	$8.27	$5.03	$12.84
Lost productivity prior to the current admission^a^	80	5.6	3.4	1.0	7.0	$6.47	$3.98	$1.16	$8.12
Lost productivity during current admission^b^	80	3.6	3.1	2.1	4.4	$4.19	$3.58	$2.40	$5.13
Lost productivity for caregivers for patients < 5 years	31	7.9	6.1	4.2	8.4	$9.18	$7.11	$4.81	$9.76
Lost productivity prior to the current admission^a^	31	4.6	2.8	1.4	5.1	$5.34	$3.29	$1.59	$5.94
Lost productivity during current admission^b^	31	3.3	3.1	2.1	4.2	$3.84	$3.63	$2.49	$4.82
Lost productivity for caregivers for patients ≥ 5 years	46	9.9	8.3	5.0	11.7	$11.48	$9.68	$5.82	$13.56
Lost productivity prior to the current admission^a^	46	6.3	3.5	1.0	7.8	$7.33	$4.06	$1.16	$8.99
Lost productivity during current admission^b^	46	3.6	3.1	1.5	5.6	$4.15	$3.55	$1.79	$6.47
Total indirect costs by wealth index									
Least poor	26	16.5	8.3	2.7	14.3	$16.67	$8.15	$0.00	$17.66
Middle	26	7.0	6.1	3.1	9.7	$4.88	$3.53	$0.00	$8.29
Poorest	28	9.0	6.0	2.1	10.2	$8.27	$3.87	$0.00	$9.43

^a^Time spent ill or providing care to sick patients prior to seeking care for current malaria episode for patients and caregivers, respectively

^b^Sum of time spent travelling to and from facility, waiting for admittance, and days spent in care

When associated caregiver costs are excluded, patients 14 years and older lost an average of 10.4 productive days (median 6.1; IQR 5.2–9.2), which was associated with an average lost productivity cost of $15.89 (median $8.31; IQR $5.97–$16.50). Likewise, excluding patient costs, caregivers alone lost a mean of 9.2 days (median 7.1; IQR 4.3–11.1) caring for and accompanying sick patients, with an associated lost productivity cost of $10.65 (median $8.27; IQR $5.03–$12.84). Caregivers accompanying patients under 5 years lost 7.9 productive days (median 6.1; IQR 4.2–8.4), compared to 9.9 productive days lost (median 8.3; IQR 5.0–11.7) for those accompanying patients 5 years and older (*p* = 0.26). Households in the least poor wealth index tertile lost an average of 16.5 productive days (median 8.3; IQR 2.7–14.3), compared to 7.0 days (median 6.1; IQR 3.1–9.7) for households in the middle wealth tertile and 9.0 days (median 6.0; IQR 2.1–10.2) for households in the poorest tertile (*p* = 0.07).

## Discussion

Despite policies aimed at providing free healthcare through the formal healthcare system [22], households in Malawi still incurred important direct and indirect costs to manage malaria episodes that resulted in admission. The average total cost of $17.48 per episode represents more than a week’s worth of income for most Malawian families; such a cost could be catastrophic to many families. Although the total costs were about 50% lower for patients treated at government facilities than the non-profit CHAM facilities, total costs at government facilities were nevertheless substantial at $12.70 per episode. While healthcare subsidies appear to be having an important effect in limiting some of the direct medical costs, relatively high medication costs, meals, and travel costs still resulted in substantial overall direct household costs at an average of $7.59 per episode. Indirect costs accounted for an additional $9.90 per episode and are likely to further compound malaria’s economic burden, particularly on less wealthy households, and will be more difficult to influence with current government policies.

Management of malaria episodes that result in admission poses a much greater economic strain on Malawian households than uncomplicated, outpatient malaria episodes. Studies in Malawi have reported direct household costs for uncomplicated malaria ranging from $1.28 to $5.52 in constant 2012 dollars [16, 17, 19]. These costs are substantially lower than the $7.59 in direct household costs that patients with a malaria episode resulting in hospitalization incurred. While inpatient malaria episodes might intuitively be expected to be more costly than outpatient episodes, relatively few previous household cost studies have examined inpatient malaria in order to quantify this difference. Additionally, while costs were highest among households in the highest wealth tertile, they remained important for households in the middle and poorest wealth tertiles at $9.82 and $14.32, respectively. Because of this, the economic burden of malaria is likely to be relatively high for the lowest income households, as has been shown elsewhere [9, 17]. This information may inform policy efforts to further subsidize the cost of inpatient malaria management and reduce the burden of severe malaria on low income households.

The high direct costs for management of malaria episodes resulting in hospitalization in Malawi are similar to those observed in Mozambique, Ghana, and Uganda for inpatient management of malaria episodes [9, 10, 33], and less than reported for severe malaria patients in Sudan where direct household costs averaged $17.20 ($22.30 in constant 2012 US dollars) for the management of severe malaria episodes [11]. This may be in part due to differences in study design; the Sudan study only reviewed costs for patients hospitalized and treated for severe or complicated malaria, whereas this study included all hospitalized patients who received anti-malarial treatment, and did not discriminate between patients with different malaria diagnoses or severity. Further studies in Malawi should prioritize assessment of household cost among patients hospitalized with a severe malaria diagnosis, as these costs are likely to be higher than those observed in this study and may be particularly catastrophic to low income households.

Patients who visited CHAM-owned facilities incurred higher costs than those who visited MoH facilities. Although CHAM facilities receive some government subsidies for their operations [2], they charge nominal service fees to cover operational costs, which results in increased direct household costs. Additionally, central medical store stock-outs sometimes force CHAM facilities to procure essential medications locally at commercial prices, the costs of which may be passed on to patients [34]. Indeed, patients in this study paid an average of $4.16 for medications at CHAM facilities compared to only $1.06 at MoH facilities. This probably reflects higher costs per medication, as there were no significant differences between the type or average number of medications received at CHAM vs. MoH facilities.

The apparently higher cost of medication at CHAM facilities represents an important avenue for policy intervention; additional governmental subsidies for anti-malarial medications at CHAM facilities and increased attention to supply chain issues could reduce direct medical costs for patients visiting CHAM facilities. This is especially important given that family wealth status was not a predictor of which type of facility patients visited, so poorer families who visit CHAM facilities could benefit substantially from such policies. Additionally, continued medication subsidies should ensure that medications remain affordable at both CHAM and MoH facilities.

Despite government efforts to expand healthcare access in rural areas of Malawi [22], increasing distance from a health facility is still associated with higher care seeking costs. Travel costs accounted for an average of 48% of direct household expenditures, and households that lived less than 5 km away from the health facility incurred a 40% lower cost per episode compared to those who lived 5 km or more away. Previous studies have shown that increased costs limit treatment seeking behavior in harder to reach households (those more than 5 km away from health facilities) [19, 33], and may account for substantial delays in accessing care [35]. Distance to a health facility and associated travel costs may, therefore, constitute a major financial burden for low income families as well as a significant barrier to prompt care-seeking, which could in turn contribute to negative health outcomes.

This study has several limitations. Because it was conducted as part of a larger study, it was not explicitly powered to examine cost outcomes. The retrospective study design introduced the possibility of recall bias, as patients may not have accurately remembered prices incurred for malaria treatment at previous health facilities, although cost information collected was limited to the current malaria episode in order to minimize recall bias. Additionally, the lost costs associated with school fees for absent days of school were not included in the indirect cost estimates, as data on school fees were not collected. Thus, the costs presented here may underestimate the true household costs of inpatient malaria episodes.

Complete malaria episode information was only available for patients who were discharged the day of the survey. Thus, the cost estimates presented here may underestimate the true overall costs. However, total costs for patients who were discharged the day of the interview compared to those who were not yet discharged were not significantly different. Furthermore, patients were not followed longitudinally. Thus, even for patients discharged the day of the survey, information on after-care, including productive time lost by patients and their caregivers during recovery, and any recrudescence and related costs were not collected. Again, this likely resulted in underestimation of overall household costs associated with malaria episodes. Finally, the findings may not be generalizable to other settings where malaria care subsidies and cost drivers vary substantially.

In settings such as Malawi where health care is heavily subsidized but direct costs remain relatively high, policy efforts to address both medical and non-medical drivers of these costs are crucial. Direct non-medical household costs may be mitigated by subsidizing meals and travel for families under a certain income level through the use of cash transfers or establishing health equity funds. Community health insurance schemes may also address the cost of care-seeking for acute febrile illness episodes. Meanwhile government subsidies should be continued and strengthened to ensure medications, consultations, and other direct medical costs remain affordable at both CHAM and MoH facilities. This is especially important given that the costs of inpatient malaria treatment might otherwise be catastrophic for the poorest families, and out of pocket costs are a major barrier to malaria care seeking among poorer households [36]. With 71% of the population of Malawi living below the poverty level, such policies could help reduce the disproportionate economic burden of malaria care on low-income households, which could in turn encourage prompt care seeking [15]. As delayed care is associated with more severe disease and poorer outcomes [37], this could reduce morbidity and mortality associated with malaria. Additional subsidies for low-income patients who visit CHAM-owned facilities may also reduce the disproportionate economic burden of malaria on poorer families.

Indirect costs may be addressed through continued malaria prevention and education efforts. With more than 3 million malaria cases every year in Malawi, the economic costs due to productive days lost for patients and their caregivers are staggering, making prevention campaigns a good investment both for public health and economic growth [1, 38]. Expanding community health worker programs and enhanced communication and education efforts may also limit costs through encouraging patients to seek care earlier in their malaria episode when care is likely to be more effective, leading to shorter duration of care and better outcomes [37]. Ultimately, prevention of malaria episodes remains the best means to avoid these costs.

## Conclusion

The findings of this study contribute to the evidence highlighting the substantial economic burden of malaria episodes resulting in hospitalization on households. While direct expenditures are an important component of household costs, indirect costs due to lost productivity magnify the economic burden of inpatient malaria management on families, and costs were notably higher for patients from hard-to-reach households and for those who visited CHAM compared to MoH facilities. As perceived and actual costs of care are important dimensions of access, minimizing these costs is crucial to protect households from potentially catastrophic health expenditures, and ensure that all patients receive prompt and effective care.
